# Resonant Raman scattering of few layers CrBr_3_

**DOI:** 10.1038/s41598-024-57622-w

**Published:** 2024-03-29

**Authors:** Łucja Kipczak, Arka Karmakar, Magdalena Grzeszczyk, Róża Janiszewska, Tomasz Woźniak, Zhaolong Chen, Jan Pawłowski, Kenji Watanabe, Takashi Taniguchi, Adam Babiński, Maciej Koperski, Maciej R. Molas

**Affiliations:** 1 Faculty of Physics, Institute of Experimental Physics, University of Warsaw, 02-093 Warsaw, Poland; 2 Institute for Functional Intelligent Materials, National University of Singapore, Singapore, 117544 Singapore; 3 Department of Semiconductor Materials Engineering, Faculty of Fundamental Problems of Technology, Wrocław University of Science and Technology, 50-370 Wrocław, Poland; 4 Faculty of Physics, Institute of Theoretical Physics, University of Warsaw, 02-093 Warsaw, Poland; 5 School of Advanced Materials, Shenzhen Graduate School, Peking University, Shenzhen, 518055 China; 6 Research Center for Electronic and Optical Materials, National Institute for Materials Science, 1-1 Namiki, Tsukuba, 305-0044 Japan; 7 Research Center for Materials Nanoarchitectonics, National Institute for Materials Science, 1-1 Namiki, Tsukuba, 305-0044 Japan; 8 Department of Materials Science and Engineering, National University of Singapore, Singapore, 117575 Singapore

**Keywords:** Condensed-matter physics, Two-dimensional materials

## Abstract

We investigate the vibrational and magnetic properties of thin layers of chromium tribromide (CrBr_3_) with a thickness ranging from three to twenty layers (3–20 L) revealed by the Raman scattering (RS) technique. Systematic dependence of the RS process efficiency on the energy of the laser excitation is explored for four different excitation energies: 1.96 eV, 2.21 eV, 2.41 eV, and 3.06 eV. Our characterization demonstrates that for 12 L CrBr_3_, 3.06 eV excitation could be considered resonant with interband electronic transitions due to the enhanced intensity of the Raman-active scattering resonances and the qualitative change in the Raman spectra. Polarization-resolved RS measurements for 12 L CrBr_3_ and first-principles calculations allow us to identify five observable phonon modes characterized by distinct symmetries, classified as the A$_\text {g}$ and E$_\text {g}$ modes. The evolution of phonon modes with temperature for a 16 L CrBr_3_ encapsulated in hexagonal boron nitride flakes demonstrates alterations of phonon energies and/or linewidths of resonances indicative of a transition between the paramagnetic and ferromagnetic state at Curie temperature ($T_\text {C} \approx 50$ K). The exploration of the effects of thickness on the phonon energies demonstrated small variations pronounces exclusively for the thinnest layers in the vicinity of 3–5 L. We propose that this observation can be due to the strong localization in the real space of interband electronic excitations, limiting the effects of confinement for resonantly excited Raman modes to atomically thin layers.

## Introduction

The recent discovery of magnetism in two-dimensional van der Waals (vdW) materials at the limit of atomically thin monolayers opens capabilities to study the fundamental aspects of magnetism at varied dimensionalities. From the practical perspective, the realization of magnetism down to monolayers pushes the limits of device minaturization, which becomes increasingly relevant for the development of spintronics^1,2^, valleytronics, and nanoelectronics^3–5^. The family of layered magnetic materials (LMMs) grows rapidly motivated by the search for materials that host stable magnetic orders under a variety of physical conditions, including temperature^6–8^, magnetic or electric field^7,9,10^, and pressure^11^. These intense research efforts lead to theoretical predictions of hundreds of LMM systems, of which dozens have been synthesized and characterized. The largest groups of LMMs include di- and trihalides (*e*.*g*., CrBr_3_ and CrI_3_)^12,13^, transition metal dichalcogenides (*e*.*g*., 1T-VS$_2$)^14,15^, tri- and tetrachalcogenides (*e*.*g*. FePS_3_ and CrPS$_4$)^16–21^, and metal-chalcogene-halides (*e*.*g*., CrSBr)^22,23^.

Herein, we investigate the vibrational properties of CrBr_3_, which belongs to the family of chromium trihalides (CrX_3_, X = I, Br, Cl). These three materials exhibit an intralayer ferromagnetic coupling within a monolayer, but they differ in the easy-axis magnetization exhibiting in-plane (CrCl_3_)^24,25^ or out-of-plane spin orientations (CrBr_3_^26,27^ and CrI_3_^28,29^). In their bulk form, two types of magnetic coupling between consecutive layers are apparent, *i*.*e*., ferromagnetic in CrBr_3_^26,27^ and antifferomagnetic in CrCl_3_^24,25^ and CrI_3_^28,29^. However, the interlayer couplings in CrX_3_ materials were found to be more complex, displaying thickness- and/or gate-dependent ferromagnetic or antiferromagnetic characteristics^3,12,28–30^. For CrBr$_\text {3}$, the interlayer coupling is mostly ferromagnetic, however, contributions from antiferromagnetic coupling have been observed in bulk crystals via inspection of magneto-resistance in vertical tunneling junctions^31^.

From the perspective of studying lattice dynamics, the Raman scattering (RS) technique has been established as a pivotal tool to uncover the physics of the vibrational and electronic properties of layered vdW materials, as well as a method to identify the exact number of layers^32–35^. Particularly, the RS characterization of CrBr_3_ revealed a chiral character of the lattice excitations in a rather complex elementary cell^8^. These phonon modes are coupled to the magnetic order, predominantly driven by modifications of the exchange coupling between neighboring Cr${^{3+}}$ ions due to vibrational motion of the lattice^36^.

In this work, we further explore the interplay between vibrational, electronic, and magnetic characteristics of thin CrBr_3_ layers through systematic polarization-resolved RS characterization as a function of the energy of laser excitation, temperature, and the thickness of the magnetic layers. We concluded that for 12 L CrBr_3_ 3.06 eV excitation energy creates resonant conditions characterized by enhanced RS efficiency at the cryogenic temperature *T* = 5 K. Five Raman peaks were identified in the polarization-sensitive resonant RS spectra, characterized by the A$_\text {g}$ (out-of-plane) and E$_\text {g}$ (in-plane) symmetries, as confirmed by first-principles calculations. Curie temperature ($T_\text {C}$) for CrBr_3_ is determined to be $\sim$50 K from the measured sudden energy redshift of the Raman resonances triggered by the increase of temperature above the critical point, at which the ferromagnetic order collapses in favor of the paramagnetic response. Moreover, the effect of thicknesses on the phonon energies reveals a notable change in phonon energy only for the thinnest layers (3–5 L CrBr_3_), indicative of the strong spatial localization of excitons^37^ which are expected to be responsible for the resonant characteristics of the enhanced vibrational response.

## Results and discussion

**Figure 1:**
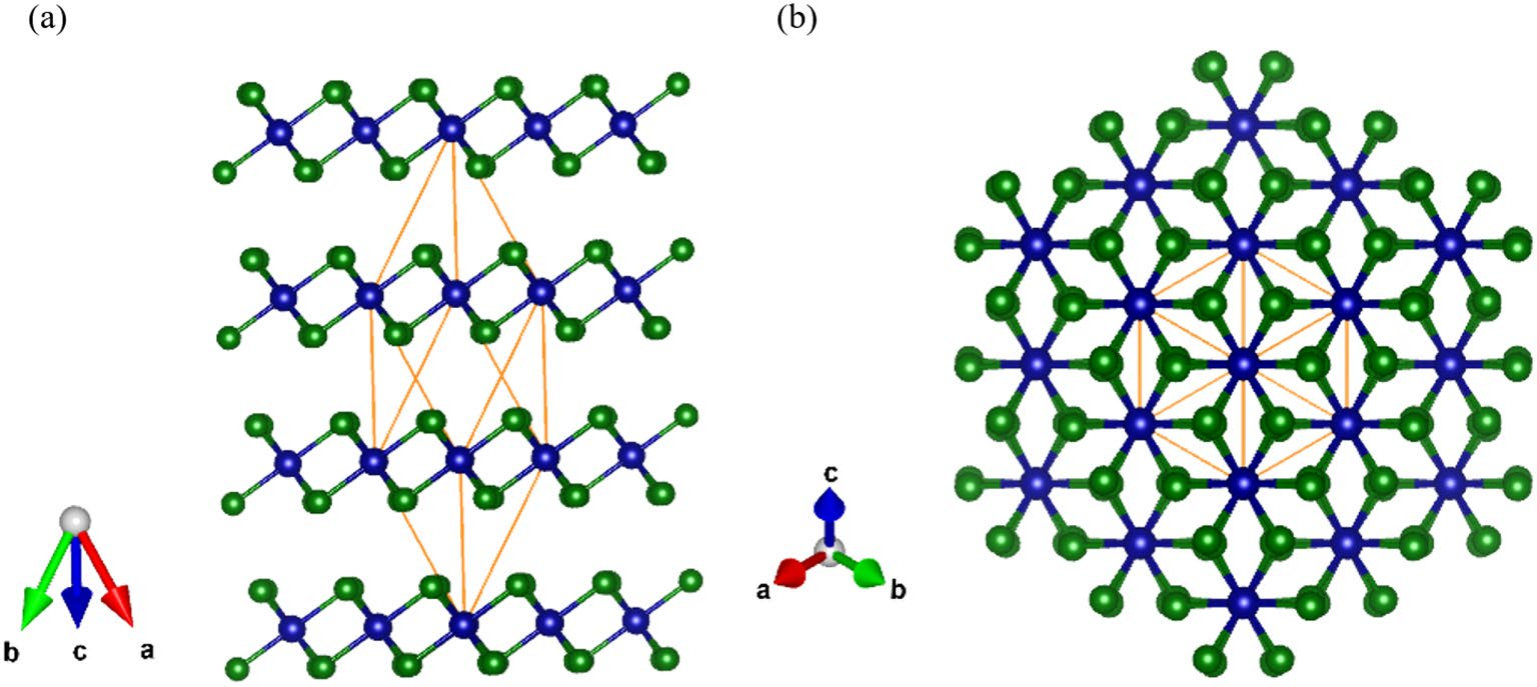
The schematic representation of (**a**) a side view and (**b**) a top view the atomic structure of the CrBr_3_ crystal in rhombohedral configuration. The orange parallelepiped represents the unit cell, which consists of three molecular layers including two Cr atoms (blue) and six Br atoms (green) per layer.

### Crystal structure of CrBr_3_

**Figure 2:**
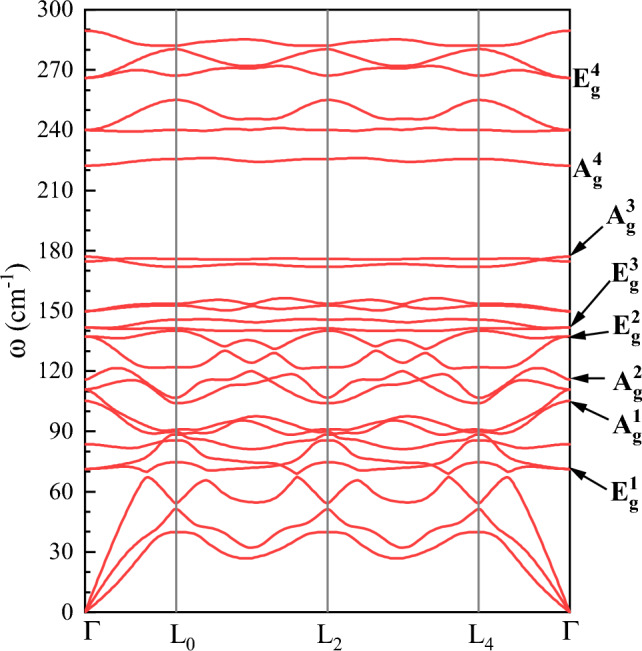
Phonon dispersion of bulk CrBr_3_ crystal in rhombohedral primitive cell with ferromagnetic order. The $\Gamma$ point corresponds to the center of the Brillouin zone.

CrBr_3_ crystallizes in a rhombohedral BI_3_ structure of R${\bar{3}}$ symmetry. The schematic representation of the atomic structure of the CrBr_3_ crystal is shown in Fig. 1. Within each layer, Cr atoms form a hexagonal crystal lattice, with each atom bonded to its six neighboring Br atoms, forming an octahedral configuration. The CrBr_3_ layers are stacked along the *c* axis and are held together by vdW interactions^38–40^. CrBr_3_ crystal exibits the rhombohedral arrangement at low and ambient temperatures^36,41^, unlike its counterparts, CrI_3_ and CrCl_3_, which undergo a structural transition from the monoclinic AlCl_3_ phase of C2/m symmetry at ambient temperature towards rhombohedral R${\bar{3}}$ structure at low temperature. The transition was identified to occur at 210 K and 240 K for CrI_3_ and CrCl_3_, respectively^24,42^. The rhombohedral primitive cell belongs to space group no. 148 with Cr and Br atoms in *6c* and *18f* Wyckoff positions, respectively. It gives rise to eight Raman-active modes, classified as: $\Gamma$ = 4A$_{g}$ + 4E$_{g}$, where the E$_{g}$ modes are doubly degenerate^36,41,43^. We calculated the dispersion of the phonon modes for bulk CrBr_3_ in the presence of ferromagnetic order in the density functional theory (DFT) framework, as illustrated in Fig. 2. The superscripts in the labels of the peaks describe additional numbering due to their increased Raman shift. The optimized lattice constant of rhombohedral cell, $a=7.128$ Å, corresponds to $a=6.352$ Å in hexagonal cell, in good agreement with the experimental value of 6.302 Å^41^.

**Figure 3:**
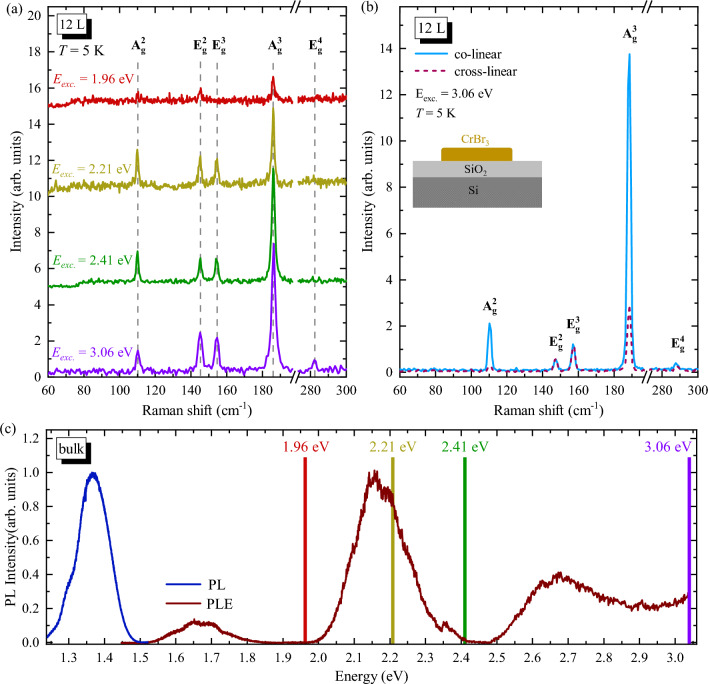
(**a**) Raman Scattering (RS) spectra of 12 L of CrBr_3_ measured at 5 K with different excitation energies: 1.96 eV, 2.21 eV, 2.41 eV and 3.06 eV, using excitation power 50 $\upmu \,W$. Spectra were shifted vertically for better visual representation. (**b**) Low-temperature (*T* = 5 K) RS spectra of the same flake, but at different spatial location, measured in co- (light-blue) and cross-linear (dashed dark pink) configurations of polarized light using 3.06 eV excitation energy and power 50 $\upmu$ W. Insert represents a schematic illustration of the sample cross-section. (**c**) Low-temperature (*T* = 1.6 K) PL (dark-blue) and PLE (brown) spectra of bulk CrBr_3_. Lines in different colors have been plotted on the PLE spectra to represent the laser excitation energies in RS experiments.

### Resonant conditions of Raman scattering in CrBr_3_

The representative low temperature ($T=5$ K) unpolarized RS spectra of a mechanically exfoliated 12 L CrBr_3_ deposited on Si/SiO$_2$ substrate are demonstrated in Fig. 3a. We comparatively inspect the dependence of the RS resonances on the laser energy, utilizing 1.96 eV, 2.21 eV, 2.41 eV, and 3.06 eV excitations. There are five distinct RS peaks in the spectra, and these peaks can be attributed to the in-plane E$_\text {g}$ and out-of-plane A$_\text {g}$ modes^8,36,41,43,44^. Specifically, four phonon modes, apparent at 110 cm$^{-1}$ (A$^2_\text {g}$), 146 cm$^{-1}$ (E$^2_\text {g}$), 156 cm$^{-1}$ (E$^3_\text {g}$), and 187 cm$^{-1}$ (A$^3_\text {g}$), are observed under all the used excitation energies, while the fifth one at 282 cm$^{-1}$ (E$^4_\text {g}$) is seen exclusively when excited with a 3.06 eV energy. DFT calculations yield frequencies of 110 cm$^{-1}$, 137 cm$^{-1}$, 142 cm$^{-1}$, 177 cm$^{-1}$ and 266 cm$^{-1}$, respectively, which agree well with the experimentally determined values. We also measured polarization-resolved Raman spectra of the 12 L CrBr_3_ flake in two configurations of linearly polarized excitation and detection: co- (solid light blue curve) and cross-linear (dashed dark pink curve), see Fig. 3b. As is shown in Fig. 2, phonon modes in CrBr_3_ at the $\Gamma$ point exhibit E$_\text {g}$ and A$_\text {g}$ characters. Consequently, the scattering resonances are apparent in both co- and cross-linear configurations (E$_\text {g}$) or only in the co-linear one (A$_\text {g}$). The spectra, resolved by the linear polarization demonstrated in Fig. 3b, are consistent with the aforementioned symmetry analysis of the phonon modes. Note that the observation of the A$^3_\text {g}$ mode in the cross-linear alignment likely originates from the resonant excitation conditions of RS. The coupling with an exciton can modify the polarization properties of the scattering resonances in the RS experiment.

The intensities of the phonon resonances change with the variation of the excitation energy, see Fig. 3a. The smallest intensity of all observed Raman modes occurs at an excitation energy of 1.96 eV. Intermediate intensities were observed for excitation energies of 2.21 eV and 2.41 eV. Finally, the highest intensities for the E$^{2}_{g}$, E$^{3}_{g}$, and A$^{3}_{g}$ modes are seen under excitation of 3.06 eV, but simultaneously the A$^{2}_{g}$ intensity is smaller as compared to the 2.21 eV and 2.41 eV excitations. A similar variation in the intensity of the RS spectra under different excitations was reported in Ref.^36^, where the Raman spectra of the CrBr_3_ monolayer were investigated using two different excitation energies, *i*.*e*., 1.65 eV and 2.09 eV. Similar trend representing the modifications of the E$^{4}_{g}$ mode intensity was indicative of a strong dependence on the applied excitation energy.

It is well established that the intensity of the RS signal in vdW materials is significantly dependent on the excitation energy^45^. In the simplest approach, the resonant conditions of RS, related to the electron-phonon coupling in a material, occur when the excitation energy of the RS processes is in the vicinity of a given transition (*e*.*g*. electronic or excitonic) in the material^46^. To identify possible transitions involved in the resonant conditions of RS in CrBr_3_, we measured photoluminescence excitation (PLE) spectrum by probing the broad photoluminescence (PL) band of bulk CrBr_3_ under continuously modified laser energy, see Fig. 3c. The PL spectrum of the CrBr_3_ bulk is characteristic for the molecular crystal, where excitons are localized on individual molecules (Frenkel type) and its recombination follows the Frank-Condon principle, as previously reported in Ref.^37^. The corresponding PLE spectrum is composed of three broad transitions centered at about 1.68 eV, 2.16 eV, and 2.65 eV, and an increase in the absorption strength can be observed at energies higher than about 2.9 eV. These peaks coincide with three significant resonances apparent at energies of about 1.7 eV, 2.2 eV, and 3.0 eV, reported in experimental low temperature ($T\sim 2-4$ K) absorption spectra of CrBr_3_^26,27,47–50^ and in the corresponding theoretical predictions of the imaginary part of the dielectric function^51^. The colored lines indicated in the PLE spectrum represent the excitation energies of lasers used for RS measurements. In particular, there are no absorption resonances at excitations of 1.96 eV and 2.41 eV, while the 2.21 eV and 3.06 eV laser energies occur at spectral ranges of significant absorption strength. Except for 2.41 eV energy, the PLE spectrum of the CrBr_3_ bulk can qualitatively explain the resonant excitation conditions of Raman scattering, suggesting the influence of the excited states of the Frenkel exciton on the electron-phonon coupling in CrBr_3_. The most challenging is the qualitative description of the Raman spectrum intensity under 2.41 eV excitation. The studies of the resonant Raman conditions in thin layers of transition metal dichalcogenides reveal that resonant cases can not be explained only by coincidence between the laser energy and the maximum of electronic density states^52^, but it requires sophisticated calculations of impacts of all transitions from the whole Brillouin zone to the electron-phonon coupling^53^. We believe that our results will motivate the development of theoretical models to describe the exciton-phonon interaction in chromium trihalides.

**Figure 4:**
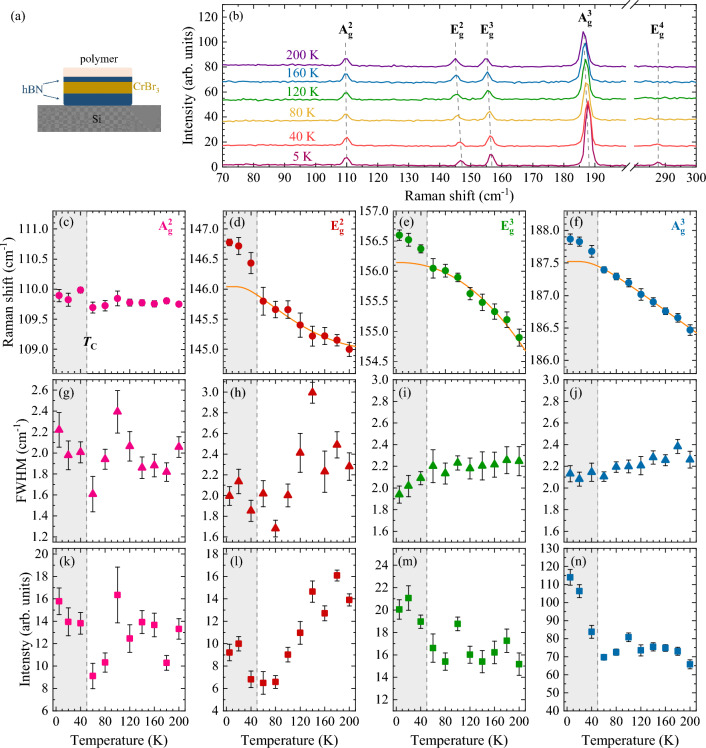
(**a**) Schematic representation of the investigated sample. The 16 L flake of CrBr_3_ was encapsulated in hBN and coated with an optically inactive polymer and placed on silicon substrate. (**b**) Temperature evolution of RS spectra of CrBr_3_ measured at selected temperature between 5 and 200 K under the 3.06 eV excitation and *P*=100 $\upmu$ W. Temperature dependence of (**c**)–(**f**) Raman shifts, (**g**)–(**j**) FWHMs and (**k**)–(**n**) integrated intensities obtained of the A$^{2}_{\text {g}}$, E$^{2}_{\text {g}}$, E$^{3}_{\text {g}}$, and A$^{3}_{\text {g}}$ phonon modes. The errors are indicated by black bars. The shaded grey rectangle represents the ferromagnetic region, while the border delineates the Curie temperature ($T_{\text {C}}$).

### Temperature effect on phonon modes in CrBr_3_

Most LMMs undergo different types of degradation, such as photocatalysis and photochemical or photothermal oxidation, which are substantially dependent on various factors such as light exposure and temperature^54–60^. It has been reported that CrCl_3_ is the most stable CrX_3_ material in ambient conditions^57,61^, while the degradation processes increase for CrBr_3_^59^, and CrI_3_ is the most unstable chromium trihalide^54^ During our investigation, we also observed that exfoliated thin CrBr_3_ flakes on Si/SiO$_2$ substrates are subjected to the degradation process under laser illumination, particularly at higher temperatures, close to room temperature. Therefore, we performed the temperature-dependent RS spectra on 16 L flake encapsulated in hBN layers, and the structure was also covered by a thin layer of optically inactive polymer to add additional protection (see section “Methods” for details) against degradation (see Fig. 4a). The temperature evolution of the Raman spectra of the CrBr_3_ flake is presented in Fig. 4b. Note, that we were able to carry out the measurements only up to 200 K due to the degradation caused by the elevated temperature. There are three effects of the increased temperature on a given Raman peak that are revealed: red shift of energy, broadening of linewidth, and variation of intensity. To investigate the temperature effect in more detail, we fitted the observed phonon modes with Lorentzian functions. The obtained temperature evolutions of the peak energies, full widths at half maximum (FWHMs), and intensities for four phonon modes, *i*.*e*., A$^{2}_{\text {g}}$, E$^{2}_{\text {g}}$, E$^{3}_{\text {g}}$, A$^{3}_{\text {g}}$, are shown in Fig. 4c–f, (g)–(j), and (k)–(n), respectively.

While the Raman shift of the A$^{2}_{\text {g}}$ mode is relatively insensitive to temperature changes, three other modes (E$^{2}_{\text {g}}$, E$^{3}_{\text {g}}$, and A$^{3}_{\text {g}}$) gradually redshift with increasing temperature. Especially, in between 40 and 60 K the energies undergo significant redshifts, followed by nearly linear decreases at higher temperatures. These abrupt reductions were observed in the literature and identified as a fingerprint of the transition from the ferromagnetic phase at low temperature to the paramagnetic phase at higher temperatures^8,36,41,43^. We estimate the Curie temperature ($T_\text {C}$) for CrBr_3_ to be around 50 K. The determined value of $T_\text {C}$ is in very good agreement with the recent result obtained for the exfoliated CrBr_3_ flake (47 K^8^), but is much bigger value as compared to the one for as-grown crystal (33 K^27^ and 27 K^41^) or in a monolayer limit (25 K^36^). The $T_\text {C}$ difference between the exfoliated flakes and the as-grown crystal of CrBr_3_ can be explained by the influence of stress on the magnetic interactions between atoms, which probably leads to a modulation of the transition temperature between the ferromagnetic and paramagnetic phases^62^.

Typically, the temperature evolution of phonon energies can be described using the anharmonic model proposed in Ref.^63^, which reads:

1 $$\omega (T) = \omega_{0} + A\left(1+ {\frac{2}{{e^{x} + 1}}} \right) + B\left( {1 + \frac{3}{{e^{y} - 1}} + \frac{3}{{(e^{y} - 1)^{2} }}} \right)$$

where the *ω*_0_, *A*, and *B* are fitting parameters, $x = \frac{\hslash \omega _{0}}{2k_{B}T}$, $y = \frac{\hslash \omega _{0}}{3k_{B}T}$ and $\omega _{0}$+*A*+*B* is the phonon frequency at 0 K.

As can be seen in Fig. 4d–f, this model can characterize the phonon temperature dependences, but only for temperature higher than $T_\text {C}$. At $T<T_\text {C}$ the phonon energies are significantly larger than those predicted by the model. The reason for this aspect is the spin-phonon coupling caused by ionic motions^41^. The spin-phonon coupling coefficient can be given by the formula^8,64–66^:

2 $$\begin{aligned} \omega (T) \sim \omega _{anh}(T) + \lambda \langle S_{i} \cdot S_{j} \rangle \end{aligned}$$

where $\omega (T)$ represents the measured phonon energy, $\omega _{anh}(T)$ denotes the phonon energy without spin-phonon coupling, $\lambda$ denotes the coupling strength, $\langle S_{i} \cdot S_{j} \rangle$ is the spin-spin correlation function of neighboring spins, and the values of $S_{i}$ and $S_{j}$ are 3/2^8,67,68^. The obtained $\lambda$ values for the $\omega _{anh}$ (60 K) are 0.43 cm$^{-1}$, 0.24 cm$^{-1}$, and 0.21 cm$^{-1}$ correspondingly for the E$^{2}_{g}$, E$^{3}_{g}$, and A$^{3}_{g}$ modes. These values agree with the previously reported data for CrBr_3_ at 70 K^8^.

We also observe a highly disordered dependence of the FWHMs on temperature for the A$^{2}_{\text {g}}$ and E$^{2}_{\text {g}}$ modes, whereas the corresponding dependences for E$^{3}_{\text {g}}$, and A$^{3}_{\text {g}}$ are monotonic with their slow growth at higher temperatures. There is no significant signature of the ferro-to-paramagnetism transition in the temperature evolutions of the FWHMs, in contrast to the results reported in Refs.^8,41^. Kozlenko et al.^41^ demonstrated that the mode linewidths are reduced within the ferromagnetic phase, reaching a minimum at $T_\text {C}$ and are followed by their monotonic growth at higher temperatures. On the other hand, Yin et al.^8^ showed similar linewidth behaviors, but the minimum of phonon FWHMs was identified at lower temperature than $T_\text {C}$. Our results accompanied by the literature^8,41^ indicate that the analysis of the phonon linewidths in CrBr_3_ is burdened with greater uncertainty in assigning the Curie temperature.

**Figure 5:**
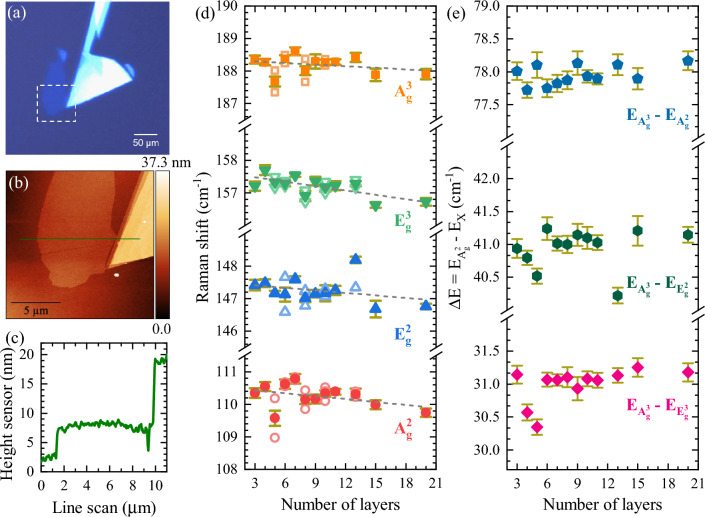
(**a**) Optical image of 8 L flake of CrBr_3_. Dashed white cube corresponds to an area scanned using atomic force microscopy (AFM). (**b**) AFM image of the flake. A horizontal line represents the (**c**) height profile of this flake. (**d**) Raman shift as a function of number of layers. The solid points represents the average Raman shift values, while the open, lighter points correspond to values of Raman shift for each layer. The errors are indicated by dark yellow bars. (**e**) Energy difference between the most prominent A$^{2}_{g}$ Raman mode and the remaining modes. The errors are indicated by dark yellow bars.

The analysis of the temperature dependence of the phonon intensities is the most intriguing, as there can be two competing transition processes: non-resonant versus resonant conditions of RS excitation and ferro- versus paramagnetic order. The intensities of the A$^{2}_{\text {g}}$ and E$^{3}_{\text {g}}$ modes exhibit a seemingly stochastic distribution, while the ones corresponding to the E$^{2}_{\text {g}}$ and A$^{3}_{\text {g}}$ are described by more organized trends. Particularly, the A$^{3}_{\text {g}}$ intensity is reduced of about 40$\%$ between 5 and 50 K, and then its intensity stabilizes at an almost fixed level. This result indicates that the intensity can also be used to distinguish the transition temperature between different magnetic phases, as reported for other LMMs, *e*.*g*., CrSBr^69^ and Cr$_2$Ge$_2$Te$_6$^70^.

### Thickness-dependent Raman scattering spectra of CrBr_3_

The final part of our work is devoted to the examination of the thickness influence on the RS spectra of thin CrBr_3_ exfoliated on the Si/SiO$_2$ substrates. We investigate 25 flakes with thicknesses ranging from 3 to 20 L. Knowing that CrBr_3_ degradation is quite slow in the ambient atmosphere, compared to its photocatalytic oxidation^59^, the flakes thicknesses were determined using the AFM technique and then subjected to Raman spectroscopy characterization. Figure 5a,b show the optical and atomic force microscopy (AFM) images of the selected CrBr_3_ flake, respectively. Its thickness is estimated to be about 5 nm (see Fig. 5c), which corresponds to 8 L (the experimental thickness of a single layer of CrBr_3_ equals 6 Å^50^). Figure 5d presents the thickness dependence of the Raman shifts of four phonon modes. The open points represent the phonon energies measured for the individual flakes, characterized by different thickness whereas the solid points denote the mean values calculated for a particular layer thickness. A discernible tendency of the phonon energies as a function of the layer thickness is apparent in the figure. However, the dependence of the Raman shifts, which ranges only from 0.4 to 0.8 cm$^{-1}$ in the investigated range of thicknesses, is hidden by their substantial random variations also within the same number of layers. To better visualize the thickness effect, Fig. 5e illustrates the energy differences between the most prominent A$^{3}_{g}$ peak and others (A$^{2}_{\text {g}}$, E$^{2}_{\text {g}}$, and E$^{3}_{\text {g}}$). The observed dependences can be divided into two groups: 3–5 L and 6–20 L. While an evident reduction in the energy difference between the A$^{3}_{\text {g}}$ and E$_{\text {g}}$ (E$^{2}_{\text {g}}$ and E$^{3}_{\text {g}}$) modes is observed when the number of layers increases from 3 to 5 layers, there is no similar behavior for the two A$_{\text {g}}$ peaks (A$^{2}_{\text {g}}$ and A$^{3}_{\text {g}}$). For the 6–20 L range, all the energy differences between the phonon modes are not affected by the thickness and stay almost at the same level. Our results, shown in Fig. 5, are in agreement with the thickness dependences previously observed for thin layers of transition metal dichalcogenides, *e*.*g*. MoS$_2$^32,71^, MoTe$_2$^33,72^, and ReSe$_2$^34^. The energy differences between the A$_{\text {g}}$ and E$_{\text {g}}$ modes are established to be the result of the interaction of the interlayer and surface effects^71,72^. In the case of thin layers of other CrX_3_, *i*.*e*. CrCl_3_^73,74^ and CrI_3_^6^, the thickness evolutions of phonon modes were also investigated. For CrCl_3_, a significant difference in Raman shifts between 1 - 3 L and the bulk was theoretically predicted^73^, but was not observed in experiments^74^. Although substantial shifts in magnon modes as a function of layers thicknesses were reported in CrI_3_ in the limit below 12 layers^6^. Or results may be indicative of the strong spatial localization of excitons^37^, which are expected to be responsible for the resonant characteristics of the enhanced vibrational response.

## Summary

The vibrational and magnetic properties of the thin layers of CrBr_3_ were investigated using Raman scattering spectroscopy. We found that the resonant RS for CrBr_3_ is observed at low temperature under the 3.06 eV excitation, in agreement with absorption bands revealed by photoluminescence excitation spectroscopy. Furthermore, the temperature dependence of the phonon energies in a 16 L CrBr_3_ encapsulated film in hBN flakes was analyzed to determine its Curie temperature of about 50 K. Finally, it was established that the effect of thickness on the phonon energies is pronounced only for the thinnest layers in the atomically-thin regime of 3 to 5 layers of CrBr_3_.

## Methods

### Samples preparation

In the case of initial studies and thickness evolution, thin flakes of CrBr_3_ obtained by polydimethylsiloxane (PDMS)-based exfoliation from bulk crystal and then stacked on SiO$_2$/Si substrates with a thickness of 90 nm or 300 nm of SiO$_2$ using an all-dry deterministic stamping technique to avoid glue residues^75^. For temperature-dependent and photoluminescence excitation (PLE) measurements, we used samples prepared from a CrBr_3_ flake and hBN flakes that were exfoliated directly on a 285 nm SiO$_2$/Si substrate in an inert gas glovebox (O$_2$ 1 ppm, H$_2$O<1 ppm). Then, a poly(bisphenol A carbonate)/polydimethylsiloxane, referred to as a polymer stamp, was used to pick up 50 nm hBN, 10 nm CrBr_3_ and 50 nm hBN, respectively at 80$^{\circ }$C with the assistance of the transfer stage in the glovebox. The polymer was left on the structure for protection. CrBr_3_ flakes of interest were first identified by visual inspection under an optical microscope and then subjected to atomic force microscopy to obtain their thickness. Bulk CrBr_3_ crystals were purchased from HQ Graphene.

### Raman scattering spectroscopy

Raman scattering spectra were measured under laser excitations of diode lasers from the Cobolt ($\lambda$ = 405 nm (3.06 eV), $\lambda$ = 515 nm (2.41 eV), $\lambda$ = 561 nm (2.21 eV)), and of He-Ne laser from the Thorlabs ($\lambda$ = 633 nm (1.96 eV)) on samples placed on a cold finger of a continuous-flow cryostat, which allows experiments to be carried out as a function of temperature from about 5 K to room temperature. The excitation light was focused by means of a 50$\times$ long-working-distance objective with a 0.55 numerical aperture (NA) producing a spot of about 1 $\mu$m diameter. The signal was collected via the same objective (backscattering geometry), sent through a 0.75 m spectrometer from the Teledyne Princeton Instruments equipped with 1800 grooves/mm, and then detected using a liquid nitrogen-cooled charge-coupled device (CCD). The laser light was filtered using longpass filters (515 nm, 561 nm, and 633 nm) or a set of Bragg-grating Notch filters (405 nm). The polarization-resolved Raman spectra were analyzed by a motorized half-wave plate and a fixed linear polarizer mounted in the detection path.

### Photoluminescence excitation technique

The PLE measurements were performed with a broadband supercontinuum light source with spectral filtering by a monochromator producing a monochromatic beam with a linewidth of about 2 nm. The power on the sample was stabilized by an electrically controlled liquid crystal followed by a linear polarized acting in a feedback loop with a photodiode monitoring the laser power entering into the probe. The CrBr_3_ sample was investigated in a dry cryogenic system at 1.6 K. The laser light was focused with a microscope objective down to a spot of about 1 $\upmu$m. The sample was mounted on a piezo-stage that allowed *x*–*y*–*z* positioning. The light emitted from the sample was collected through the same objective and dispersed by a 0.75 m spectrometer from the Teledyne Princeton Instruments equipped with 150 grooves/mm, and then detected using a CCD.

### DFT calculations

The first-principles calculations were performed within the density functional theory (DFT) in the Vienna ab-initio simulation package (VASP)^76–79^. The projector augment wave (PAW) potentials and general gradient approximation (GGA) of Pedew-Burke-Ernzerhof (PBE)^80^ with D3 van der Waals correction^81^ were used. Spin-orbit coupling was not included due to its insignificant impact on lattice relaxation and phonon dispersion in this compound, as shown by Pandey et al. in Ref.^64^. The unit cell and atomic positions have been optimized until forces on atoms were lower than 10$^{-5}$ eV/Å and stress tensor components were lower than 0.1 kbar. A plane wave basis set cutoff of 500 eV and a $\Gamma$-centered k-mesh 6$\times$6$\times$6 were sufficient to converge the lattice constants with precision of 0.001 Å. An energy tolerance of 10$^{-8}$ eV was used to in self-consistent loop. The phonon dispersion was calculated using the finite displacement method as implemented in Phonopy package^82,83^. $2\times 2 \times 2$ supercells were found to assure convergence of the phonon frequencies at $\Gamma$ point. All the calculations were performed at temperature of 0 K.
